# General considerations for online teaching practices in bioinformatics in the time of COVID‐19

**DOI:** 10.1002/bmb.21558

**Published:** 2021-07-07

**Authors:** Miguel Arenas

**Affiliations:** ^1^ CINBIO Universidade de Vigo Vigo Spain; ^2^ Department of Biochemistry, Genetics and Immunology Universidade de Vigo Vigo Spain

**Keywords:** bioinformatics, COVID‐19 pandemic, online teaching, planning for teaching, practices in bioinformatics

## Abstract

The COVID‐19 pandemic is promoting online education. In this concern, not only online teaching of wet laboratory practices is being challenging, but also online teaching of practices in bioinformatics. Here, I discuss the problematic of online teaching practices in bioinformatics and provide fundamental guidelines, especially oriented for beginner educators, that can help in the time of the pandemic.

## 1 INTRODUCTION

The online teaching of lectures during this COVID‐19 pandemic could be addressed considering the availability of diverse online frameworks and repositories.^1^ A more problematic situation is the teaching of practices in the wet laboratory, which has been transitioned to remote data analyses, virtual simulations, literature reviews, and online discussions.^2^, ^3^, ^4^ Concerning practices in bioinformatics, which are traditionally performed in a classroom with computers provided by the education center,^5^, ^6^ several aspects could complicate their online teaching. Students can have computers with different operating systems (OS), and some software of the practices could only run on specific OS or present dissimilarities running on different OS. Indeed, students could find doubts about the specific capabilities of their computers (i.e., RAM memory), and those capabilities can be needed for certain computational analyses (i.e., storage and assembly of genome data from next generation sequencing) of the practices. Here, I briefly provide recommendations, from our experience during this pandemic, for adapting in‐person to online teaching of practices in bioinformatics that could be particularly useful for beginner educators. This communication is not oriented to describe bioinformatics contents (note that bioinformatics combines a variety of disciplines such as Genetics, Biochemistry, Ecology and Biotechnology, among others, with computer science, for the analysis of biological data, modeling biological processes or writing code of bioinformatics software, among others) and can be found in other works (e.g., see Reference 7 and Data S1).

## 2 KEY TIPS FOR ONLINE EDUCATION OF PRACTICES IN BIOINFORMATICS

### 2.1 Considerations for designing the practices

In online education, including practices, students often use their own computers. In this case, the educator should ask students for the OS implemented (i.e., a week prior to the practice) and indicate the installation of software required for the practices. This can facilitate problem solving during the practices and saves time (installing software can take a long time). Ideally, the educator should consider software for the practices that is compatible with all OS and allow rapid computation. However, this is not always possible and alternatives (i.e., OS emulation or OS specific software) can be required, students should be informed in advance about it. Importantly, in the case of using online databases or servers (which are convenient because they are compatible with all OS; a list of popular online frameworks and tutorials for practices in bioinformatics is included in Data S1), the educator should consider that some students could have slow internet connection that affects waiting times of data transfer.^5^

### 2.2 Teaching during the practices

The online teaching of practices in bioinformatics should be dynamic and consider the variety of computers/OS used by the students. Providing a detailed guide of practices with information for different OS is highly recommended. Importantly, technical questions related with particular computers could slow down and alter the flux of the class and thus solving them after the class (i.e., tutorships) is recommended. Indeed, periodically interrupt the class and ask students about their learning can be convenient. After the practices, getting a feedback from the students about the online practices (i.e., a survey with pros and cons) is useful for improving next online education activities.

In summary, the online teaching of practices in bioinformatics should not only deal with the traditional limitations of online education,^8^ but also consider the variety of computer machines that the students can have to perform the practices. Thoughtfully prepared practices (including prior communication with students, prior software installation and configuration and, a detailed guide of practices with sections for different OS) are recommended to avoid delays and unexpected complications during the lessons.

## CONFLICT OF INTEREST

The author declares no conflict of interest.

## Supporting information


**Data S1**. Commonly used online frameworks and tutorials for teaching practices in Bioinformatics
