# Supplementary material for: General considerations for online teaching practices in bioinformatics in the time of COVID‐19
Source: Biochem Mol Biol Educ. 2021 Jul 7;49(5):683–4. doi: 10.1002/bmb.21558 (PMC8426940; doi:10.1002/bmb.21558)
Supplement: Supplementary file 1 — Data S1. Commonly used online frameworks and tutorials for teaching practices in Bioinformatics [file BMB-49-683-s001.docx]

*Supplementary Material*

# General considerations for online teaching of practices in Bioinformatics in the time of COVID-19

**Commonly used online frameworks and tutorials for teaching practices in Bioinformatics**

The table shows the name of the framework or tutorial, rough classification in several areas of Bioinformatics (analyses of genetic data, molecular modeling, programming Bioinformatics software and general bioinformatics), description and analyses, URL and reference (if available). NGS refers to next generation sequencing. Other online frameworks and tutorials can be found and this list presents an updated selection based on the author’s experience.

| **Online framework or tutorial** | **Classification** | **Description and analyses** | **URL** | **Reference** |
| --- | --- | --- | --- | --- |
| BLAST for beginners *(tutorial)* | Analyses of genetic data | Education tutorial of BLAST (commonly used tool for comparing nucleotide sequences) | <https://digitalworldbiology.com/tutorial/blast-for-beginners> | - |
| DNA subway *(framework)* | Analyses of genetic data | DNA sequence annotation, phylogenetic analyses, analysis NGS of data | <https://dnasubway.cyverse.org/> | - |
| Hands-On Assembly of DNA Sequencing Reads *(tutorial)* | Analyses of genetic data | Tutorial for the assembly of DNA sequencing reads (NGS data) | <https://www.asmscience.org/content/journal/jmbe/10.1128/jmbe.v18i2.1295> | [1] |
| Genomics tutorial *(tutorial)* | Analyses of genetic data | Tutorial for the assembly and analysis of NGS data using Linux and the command-line | <https://genomics.sschmeier.com/introduction.html> | - |
| Udacity *(tutorial)* | Analyses of genetic data | Introductory course on the human genome | <https://www.udacity.com/course/tales-from-the-genome--bio110> |  |
| IQ-Tree web server *(framework)* | Analyses of genetic data | Online and user-friendly phylogenetic tree reconstruction | <http://iqtree.cibiv.univie.ac.at/> | [2] |
| Phylogeny.fr *(framework)* | Analyses of genetic data | Online and user-friendly phylogenetic tree reconstruction | <http://www.phylogeny.fr/> | - |
| Primer of phylogenetic networks *(tutorial)* | Analyses of genetic data | A tutorial on the basis of phylogenetic networks | <http://acacia.atspace.eu/Tutorial/Tutorial.html> | - |
| Simulation of genetic data *(tutorial)* | Analyses of genetic data | An education tutorial on the basis simulation of genetic data | <https://journals.plos.org/ploscompbiol/article?id=10.1371/journal.pcbi.1002495> | [3] |
| MGAN *(framework)* | Analyses of genetic data | Annotation of microbial genomes | <https://mgan.geni-act.org/index.html> | - |
| Microbial Bioinformatics starter kit *(framework)* | Analyses of genetic data | A blog with tutorials on the evolutionary analyses of microbial genetic data | <https://c2-d2.github.io/hanage-lab/Microbial-Bioinformatics-starter-kit/> | - |
| Coalescent *(framework)* | Analyses of genetic data | Online frameworks for teaching the coalescent theory | <https://www.coalescent.dk/> | - |
| Datamonkey web server *(framework)* | Analyses of genetic data | User-friendly web server to analyze recombination and selection in genetic data | <http://www.datamonkey.org/> | [4] |
| Phylemon web server *(framework)* | Analyses of genetic data | A suite of online tools for molecular evolution, phylogenetics, phylogenomics and hypotheses testing | <http://phylemon.bioinfo.cipf.es/> | [5] |
| Homology modeling *(tutorial)* | Molecular modeling | Education tutorial on homology modelling, including practices and web servers | <http://www.pdg.cnb.uam.es/cursos/bcn05/Structures/3D_Practicals/P_homology/index.html> | - |
| SWISS-MODEL web server *(framework)* | Molecular modeling | A fully automated protein structure homology-modelling server | <https://swissmodel.expasy.org/> | [6] |
| Protein-ligand docking *(tutorial)* | Molecular modeling | A basic tutorial on protein-ligand docking | <https://training.galaxyproject.org/training-material/topics/computational-chemistry/tutorials/cheminformatics/tutorial.html> | - |
| Molecular Docking Tutorial *(tutorial)* | Molecular modeling | A molecular Docking Tutorial | <https://sites.ualberta.ca/~pwinter/Molecular_Docking_Tutorial.pdf> | - |
| MDWeb *(framework)* | Molecular modeling | An online user-friendly framework for molecular dynamics | <http://mmb.irbbarcelona.org/MDWeb/index.php> | - |
| Interactive Molecular Dynamics Simulation *(framework)* | Molecular modeling | An interactive framework for molecular dynamics | <https://www.ks.uiuc.edu/Research/vmd/imd/> | - |
| Introduction to molecular dynamics *(tutorial)* | Molecular modeling | Education tutorial for molecular dynamics using GROMACS | <http://sbcb.bioch.ox.ac.uk/phil/teaching/wt-prac.html> | - |
| Software Carpentry *(tutorial)* | Programming Bioinformatics software | Material for online teaching of programming Bioinformatics software | <https://software-carpentry.org/lessons/> | - |
| Cold Spring Harbor’s Programming for Biology class *(tutorial)* | Programming Bioinformatics software | Material for online teaching of programming Bioinformatics software with Python | <http://programmingforbiology.org/index.html> | - |
| Rosalind *(tutorial)* | Programming Bioinformatics software | Material for online teaching of programming Bioinformatics software with Python | <http://rosalind.info/problems/locations/> | - |
| Google's Python Class *(tutorial)* | Programming Bioinformatics software | A tutorial by Google for learning Python | <https://developers.google.com/edu/python/> | - |
| Udacity *(tutorial)* | Programming Bioinformatics software | An introductory course for learning Python | [https://www.udacity.com/course/introduction-to-python--ud1110#](https://www.udacity.com/course/introduction-to-python--ud1110) |  |
| Coursera *(tutorial)* | General Bioinformatics | It includes a courses on multiple topics of Bioinformatics | <https://www.coursera.org/courses?query=bioinformatics&languages=en> | - |
| An Online Bioinformatics Curriculum *(tutorial)* | General Bioinformatics | It includes a catalog of freely available courses on multiple topics of Bioinformatics | <https://journals.plos.org/ploscompbiol/article?id=10.1371/journal.pcbi.1002632> | [7] |
| An Introduction to Applied Bioinformatics *(tutorial)* | General Bioinformatics | A freely available and interactive tutorial on core concepts of bioinformatics | <http://readiab.org/> | - |

**References cited in the Supplementary Material**

1. Jensen PA. Hands-On Assembly of DNA Sequencing Reads as a Gateway to Bioinformatics. *J Microbiol Biol Educ*. 2017;18(2).

2. Trifinopoulos J, Nguyen L-T, von Haeseler A, Minh BQ. W-IQ-TREE: a fast online phylogenetic tool for maximum likelihood analysis. *Nucleic Acids Res*. 2016;44(W1):W232-W5.

3. Arenas M. Simulation of Molecular Data under Diverse Evolutionary Scenarios. *PLoS Comput Biol*. 2012;8(5):e1002495.

4. Weaver S, Shank SD, Spielman SJ, Li M, Muse SV, Kosakovsky Pond SL. Datamonkey 2.0: a modern web application for characterizing selective and other evolutionary processes. *Mol Biol Evol*. 2018.

5. Sánchez R, Serra F, Tárraga J, Medina I, Carbonell J, Pulido L, et al. Phylemon 2.0: a suite of web-tools for molecular evolution, phylogenetics, phylogenomics and hypotheses testing. *Nucleic Acids Res*. 2011;39(suppl_2):W470-W4.

6. Waterhouse A, Bertoni M, Bienert S, Studer G, Tauriello G, Gumienny R, et al. SWISS-MODEL: homology modelling of protein structures and complexes. *Nucleic Acids Res*. 2018;46(W1):W296-W303.

7. Searls DB. An Online Bioinformatics Curriculum. *PLOS Computational Biology*. 2012;8(9):e1002632.
